# Mammalian cells lacking either the cotranslational or posttranslocational oligosaccharyltransferase complex display substrate-dependent defects in asparagine linked glycosylation

**DOI:** 10.1038/srep20946

**Published:** 2016-02-11

**Authors:** Natalia A. Cherepanova, Reid Gilmore

**Affiliations:** 1 Department of Biochemistry and Molecular Pharmacology University of Massachusetts Medical School Worcester, Massachusetts, 01605,USA.

## Abstract

Asparagine linked glycosylation of proteins is an essential protein modification reaction in most eukaryotic organisms. Metazoan organisms express two oligosaccharyltransferase complexes that are composed of a catalytic subunit (STT3A or STT3B) assembled with a shared set of accessory subunits and one to two complex specific subunits. siRNA mediated knockdowns of STT3A and STT3B in HeLa cells have shown that the two OST complexes have partially non-overlapping roles in N-linked glycosylation. However, incomplete siRNA mediated depletion of STT3A or STT3B reduces the impact of OST complex loss, thereby complicating the interpretation of experimental results. Here, we have used the CRISPR/Cas9 gene editing technology to create viable HEK293 derived cells lines that are deficient for a single catalytic subunit (STT3A or STT3B) or two STT3B-specific accessory subunits (MagT1 and TUSC3). Analysis of protein glycosylation in the STT3A, STT3B and MagT1/TUSC3 null cell lines revealed that these cell lines are superior tools for investigating the *in vivo* role and substrate preferences of the STT3A and STT3B complexes.

Asparagine-linked (N-linked) glycosylation of proteins in the rough endoplasmic reticulum (RER) is one of the most common and fundamental post-translational protein modification reactions in eukaryotic cells^1^. Glycoproteomics analysis of murine tissues identified 6367 glycosylated asparagine residues in 2352 proteins^2^. These values likely underestimate the murine N-glycoproteome by 1.5–2 fold based upon incomplete coverage (~74% coverage) of previously identified murine glycosylation sites. The oligosaccharyltransferase (OST) catalyzes the transfer of a preassembled oligosaccharide from a dolichol pyrophosphate-linked oligosaccharide donor onto the asparagine residue of glycosylation acceptor sites or sequons (typically Asn-X-Thr/Ser, where X≠Pro, abbreviated NXT/S) in newly synthesized proteins (as reviewed in^1^,^3^). A critical question concerning N-glycosylation is how the OST glycosylates the diverse protein substrates that enter the lumen of the endoplasmic reticulum.

Mammalian cells express two OST complexes that have different catalytic subunits (STT3A or STT3B) assembled with a shared set of non-catalytic subunits (ribophorin I (Rb1), ribophorin II (Rb2), OST48, DAD1 and OST4), plus complex-specific subunits that are part of the STT3B complex (MagT1 or TUSC3)^4^,^5^. DC2 and KCP2 were initially detected as subunits of the STT3A complex^6^. Analysis of OST complexes by blue native gel electrophoresis suggests that DC2, but not KCP2, might also be present in the STT3B complex^7^,^8^. The STT3A isoform is associated with the protein translocation channel^6^,^9^, and mediates cotranslational glycosylation of NXT/S sites on nascent polypeptides^10^,^11^. The analysis of glycosylation in STT3B depleted cells indicates that the major cellular role for the STT3B complex is to maximize sequon occupancy in glycoproteins by modification of sites that are skipped by the STT3A complex^11^. Expression of both OST complexes in human cells is necessary to achieve full glycosylation of the N-glycoproteome and is essential for normal human health and development as exemplified by the recent discovery of patients with novel forms of congenital disorders of glycosylation-I (CDG-1) that are caused by mutations in the STT3A or STT3B genes that reduce, but do not eliminate, STT3A or STT3B expression^12^.

MagT1 and TUSC3 are orthologues of the yeast Ost3 and Ost6 proteins^13^. Human STT3B complexes contain either MagT1 or TUSC3 5, just as the yeast OST complex contains either Ost3 or Ost6^14^,^15^,^16^. The structures of the lumenal domains of Ost6 and TUSC3 have been solved revealing a thioredoxin fold with an active site CXXC motif that is required for function^17^,^18^. Mutations in the human MagT1 gene cause X-linked immunodeficiency syndrome (XMEN)^19^, but are no longer thought to be linked to X-linked intellectual disability (XLID)^20^. Mutations in TUSC3 cause non-syndromic autosomal recessive mental retardation (ARMR) a disease characterized by intellectual impairment^21^,^22^,^23^.

Pulse-chase labeling of human glycoproteins in siRNA treated HeLa cells revealed important information about STT3A- and STT3B-dependent glycosylation sites. Acceptor sites that are skipped at a high frequency by the STT3A complex include sequons adjacent to the signal sequence cleavage site^11^ and sequons located within the last 50 residues of proteins^24^. Closely spaced NXS acceptor sites^25^, and certain internal acceptor sites with sub-optimal sequons^26^ including a subset of acceptor sites that have internal cysteine residues (NCT/S sites) are also skipped by the STT3A complex^5^. Local sequence features that promote site skipping by the STT3A complex remain poorly defined.

Although siRNA mediated depletion of STT3A, STT3B or MagT1 has been a valuable tool for glycosylation analysis; there are several major limitations. The reduction in STT3A or STT3B protein levels seldom exceeds more than 4-fold in HeLa or CHO cells^5^,^11^,^27^ presumably due to the long half-life of OST subunits (>24 h^28^). Incomplete depletion of the STT3A or STT3B complexes almost certainly reduces the extent of protein hypoglycosylation, thereby hindering the interpretation of experimental results. Glycosylation of many NXT/S sites in glycoproteins is not reduced by siRNA-mediated depletion of STT3A or STT3B^11^,^25^. It is not clear whether these insensitive acceptor sites can be modified efficiently by either OST complex, or whether these sites are simply not susceptible to incomplete depletion of STT3A or STT3B. Notably, simultaneous siRNA mediated knockdown of STT3A and STT3B does not eliminate N-glycosylation of any substrate we have tested to date due to incomplete depletion of STT3A and STT3B. Finally, cell lines that divide less rapidly than HeLa cells will likely display lower extents of siRNA mediated depletion of STT3A or STT3B, further limiting the utility of siRNA mediated knockdowns.

Genome editing in human cells provides a powerful tool for creating knock-out cell lines. Clustered regulatory interspaced short palindromic repeat (CRISPR)/Cas9-based technology has been widely applied due to its affordability and ease of use^29^,^30^,^31^. In the present study, we describe the generation and initial characterization of HEK293-derived cell lines that do not express one of the OST catalytic subunits (STT3A or STT3B) or do not express one or both of the accessory subunits (MagT1 and TUSC3) of the STT3B complex. We have compared protein hypoglycosylation in these cell lines to siRNA treated HEK293 and HeLa cells. Human cells with null mutations in STT3A or STT3B, or complex specific accessory subunits will provide a superior model system for further investigation of the *in vivo* role of the STT3A and STT3B complexes in protein glycosylation.

## Results and Discussion

### siRNA knockdowns of OST subunits in HEK293 cells is relatively ineffective

**Figure 1:**
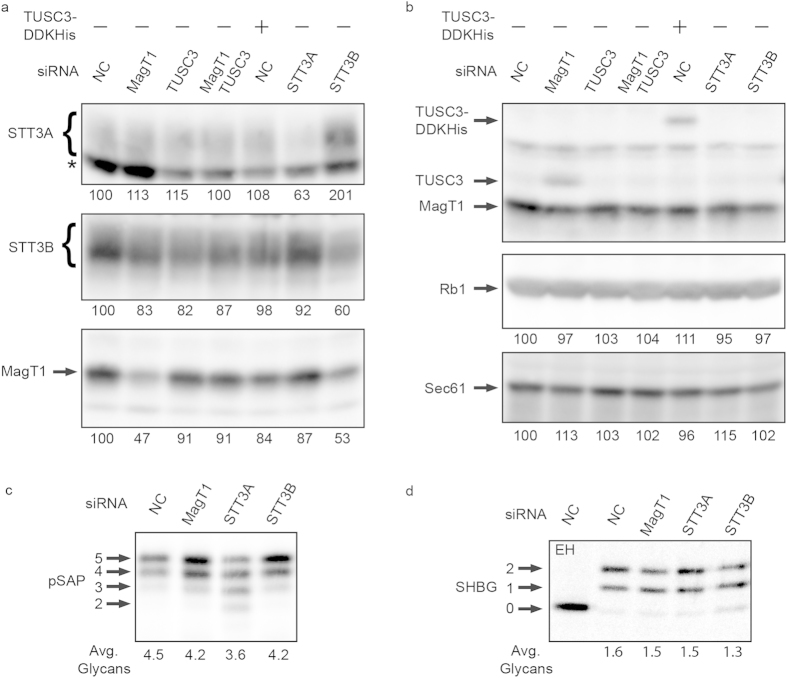
siRNA-mediated depletion of OST subunits is relatively ineffective in HEK293 cells. HEK293 cells were treated with negative control (NC) siRNA or siRNAs specific for MagT1, TUSC3, STT3A or STT3B for 72 h. (**a**,**b**) HEK293 cell extracts were resolved by SDS-PAGE and analyzed by protein immunoblotting using the specified antisera. Sec61α served as gel loading control. Expression values relative to cells treated with the NC siRNA are for the displayed image that is representative of two or more experiments. (**a**) The asterisk designates a nonspecific product recognized by the anti-STT3A sera. (**b**) Anti-TUSC3 sera crossreacts with MagT1. (**c**,**d**) Cells were transfected with expression vectors for pSAP-DDK-His (**c**) or SHBG (**d**) 48 h after siRNA treatment and pulse labeled 24 h later for either 4 min (SHBG) or 10 min (pSAP-DDKHis) and chased for either 10 min (pSAP-DDKHis) or 20 min (SHBG). Proteins were immunoprecipitated with anti-DDK or anti-SHBG sera. EH designates digestion with endoglycosidase H. Glycoforms resolved by PAGE in SDS are labeled to indicate the number of N-linked glycans. Quantified values below gel lanes (**c**,**d**) are for the displayed image that is representative of two or more experiments. (**a**–**d**) Immunoblots and phosphorimages were cropped to display the region of interest. Full-length immunoblot images and full length gel images are shown in Supplemental Fig. S2.

A 72 h treatment of HEK293 cells with our previously validated siRNAs for human STT3A, STT3B or MagT1 caused roughly a 2-fold reduction in expression levels of the targeted proteins as detected on protein immunoblots (Fig. 1a). OST subunit depletion is likely limited by the long half-life of OST subunits^28^. Expression of the catalytic subunits (STT3A or STT3B), shared OST subunits (e.g., ribophorin I), or the protein translocation channel (Sec61) was not reduced by treating cells with the MagT1 siRNA (Fig. 1a,b). Depletion of STT3B was accompanied by a reduction in MagT1 expression as observed previously in HeLa cells^5^. MagT1 mRNA is widely expressed in human tissues while TUSC3 mRNA expression is more restricted^21^,^32^. The cultured cell lines we have tested to date (HeLa, CHO, HepG2, U97, NSC-34 and human fibroblasts) express MagT1 protein, but do not express sufficient TUSC3 to readily detect by protein immunoblotting using anti-TUSC3 sera^5^, (Cherepanova, Shrimal and Gilmore, unpublished observations). Lack of detectable TUSC3 in the HEK293 cells is particularly remarkable given that the anti-TUSC3 sera cross-reacts strongly with endogenous MagT1 (Fig. 1b) due to the high amino acid sequence identity between MagT1 and TUSC3^4^,^5^. The siRNA mediated reduction in MagT1 expression in HEK293 cells, but not in HeLa cells^5^, was accompanied by the appearance of an anti-TUSC3 immunoreactive product that migrated between MagT1 and transiently expressed TUSC3-DDKHis (Fig. 1b). The protein of intermediate mobility was identified as TUSC3 based upon sensitivity to treatment with TUSC3 siRNA (Fig. 1b).

After 48 hours of siRNA treatment, the HEK293 cells were transfected with expression plasmids for prosaposin (pSAP, Fig. 1c) or sex hormone binding globulin (SHBG, Fig. 1d), the two most extensively validated substrates of the STT3A and STT3B complexes. A pulse-chase labeling experiment revealed a 20% reduction in pSAP glycosylation in cells that were treated with the STT3A siRNA (Fig. 1c). SHBG has two extreme C-terminal glycosylation sites that are glycosylated by the STT3B complex in a MagT1 dependent manner^5^,^24^. Pulse labeling of SHBG in the HEK293 cells revealed no reduction in glycosylation of SHBG in the cell treated with the MagT1 siRNA presumably due to enhanced stable expression of TUSC3. We detected a minor reduction in SHBG glycosylation in HEK293 cells treated with the STT3B siRNA (Fig. 1d). These results indicate that siRNA mediated depletion of OST subunits in HEK293 cells, unlike HeLa cells, is not efficient enough to markedly reduce glycosylation of these well-characterized substrates of the STT3A and STT3B complexes. Additional glycoprotein substrates were not tested in the siRNA treated HEK293 cells given the minor impact of the partial STT3A or STT3B depletions on pSAP and SHBG glycosylation.

### Targeting and selection of HEK293 derived cell lines

The CRISPR/Cas9 system was used to generate HEK293 derived cell lines that do not express STT3A, STT3B, MagT1 or TUSC3. Two to three CRISPR targets were selected in the MagT1, TUSC3, STT3A and STT3B genes (Supplementary Table S1). HEK293 cells were transfected with the pSpCas9(BB)-2 A-GFP vector containing sgRNA expression constructs designed to target the MagT1, STT3A and STT3B genes. After 24 h, cells were analyzed by fluorescence activated cell sorting (FACS) to obtain cells that express green fluorescent protein (GFP). Growth of single cells in 96 well plates allowed the isolation of HEK293-derived cell clones that were candidates for the desired knockout cell lines. Protein immunoblot analysis of cell extracts using antisera specific for MagT1, STT3A or STT3B was used as the primary screen to select potential knockout cell lines for further analysis. Cell lines that lacked a detectable protein immunoblot signal for the targeted OST subunit were subjected to a secondary immunoblot screen using antisera that recognize OST subunits that are specific for the STT3A complex (KCP2^6^,^8^), or the STT3B complex (MagT1 and TUSC3^5^). Protein immunoblots of the candidate MagT1(−/−) cell lines all revealed an elevated signal for TUSC3 relative to HEK293 cells. To obtain a MagT1(−/−) TUSC3(−/−) cell line, a MagT1(−/−) cell line was transfected with sgRNA constructs designed to target the TUSC3 gene. Candidate MagT1(−/−) TUSC3(−/−) cell lines were screened for expression of TUSC3 using protein immunoblots. Target sequences selected for further analysis are indicated with an asterisk in Supplementary Table 1.

### DNA sequences of targeted OST subunit genes

**Figure 2:**
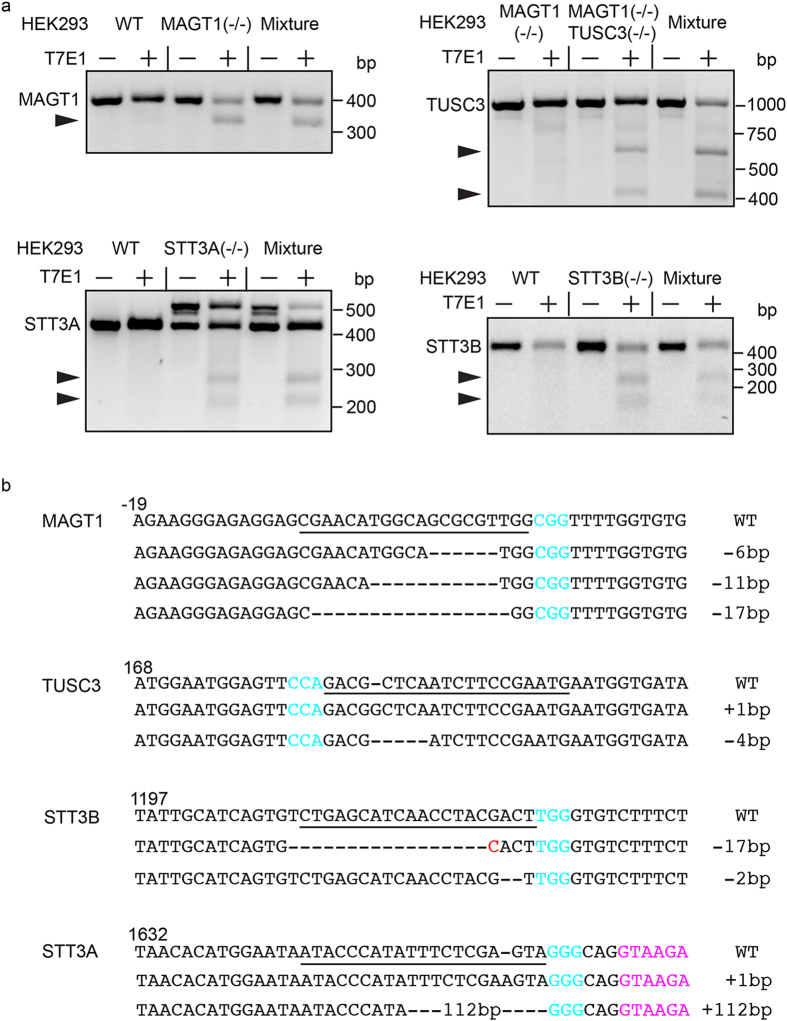
CRISPR/Cas9 genome editing in HEK293 cells. (**a**) A mismatch-specific T7E1 endonuclease assay was used to analyze DNA flanking the CRISPR target sites. Cleavage products are designated by arrowheads. Agarose gel images were cropped to display the region of interest. Full length gel images are shown in Supplemental Fig. S3. (**b**) Sequence alignment of the WT allele from control HEK293 cells and mutant alleles identified from each cell line by DNA sequencing of genomic PCR products. Nucleotide numbers are expressed relative to the initiation codon. The sequences corresponding to the 20-nt srRNA target are underligned, the 3-nt PAM is highlighted in cyan. The intron sequence adjacent to the target site in STT3A (−/−) clone is in magenta.

Potential positive clones for each knockout were expanded and subjected to several assays to characterize the knockout cell lines. Heteroduplexes derived from PCR amplicons flanking the target site were subjected to T7E1 (T7 endonuclease 1) digestion (Fig. 2a). All knockout cell lines displayed heterozygosity at the target site, since cleaved DNA fragments were produced by T7E1 digestion. PCR products from WT HEK293 cells were mixed with PCR products from the mutant, re-annealed and treated with T7E1. The samples showed similar digestion patterns further indicating that the clonal cell lines are heterozygous at the target sites.

DNA sequencing of the PCR products (15–35 sequences/cell line) was used to identify the mutations in the knockout cell lines (Fig. 2b). We did not recover the wild type OST subunit sequence from any of the knockout cell lines. It has been shown that HEK293 cells are hypotriploid^33^. The genome of HEK3293 cells contains seven copies of the MagT1 gene, three copies of the STT3A and STT3B genes and two copies of the TUSC3 gene (http://hek293genome.org/v2/). Sequencing of the PCR amplicons flanking the MagT1 target site revealed three amplification products (Fig. 2b) lacking 6 bp, 11 bp or 17 bp present at a ratio of 7:23:5 among the 35 sequences that were obtained (Fig. 2b). Sequencing of the STT3A(−/−), STT3B(−/−) and MagT1(−/−)TUSC3(−/−) cell lines identified two independent alleles for the STT3A, STT3B and TUSC3 genes (Fig. 2b). The observed number of alleles for MagT1, STT3A and STT3B could be an underestimate of the full spectrum of indels since the PCR-based method would not identify large insertions and deletions, inversions or structural aberrations^34^. For example, one of the sequenced STT3A alleles contains a large insertion (112 bp) that was intially detected by gel electrophoresis of the PCR amplification products (Fig. 2a). With one noteworthy exception, all of the identified genomic sequences contained indels that will create a frameshift mutation in the mRNA. The 6 bp in-frame deletion in the MagT1 gene will eliminate two residues from the N-terminal signal sequence. Immunoblot analysis and further functional analysis revealed that the MagT1(−/−) cells do not express MagT1 despite having this small in-frame deletion. The most likely explanation for the MagT1 null phenotype is that the MagT1 gene is located on the X-chromosome^19^,^33^, hence the allele with the in frame deletion is likely silent due to X chromosome inactivation.

Genomic sites with fewer than 5 mismatches to the sgRNA that are adjacent to a PAM site (protospacer adjacent motif, NGG) can be recognized by the sgRNA and act as off-target sites^35^. The CRISPR DESIGN webserver (http://crispr.mit.edu) was used to search for potential off-target sites for each of the sgRNAs. The three most probable off-targets sites for the sgRNAs are listed in Supplementary Table S2. PCR fragments containing potential off-target sites were amplified from WT HEK293 cell DNA as well as from the STT3A(−/−), STT3B(−/−), MagT1(−/−) and MagT1(−/−)TUSC3(−/−) cell lines. T7E1 analysis did not disclose indel mutations in any of the potential off-target sites (Supplementary Fig. S1).

### Analysis of OST protein levels in knock-out cell lines

**Figure 3:**
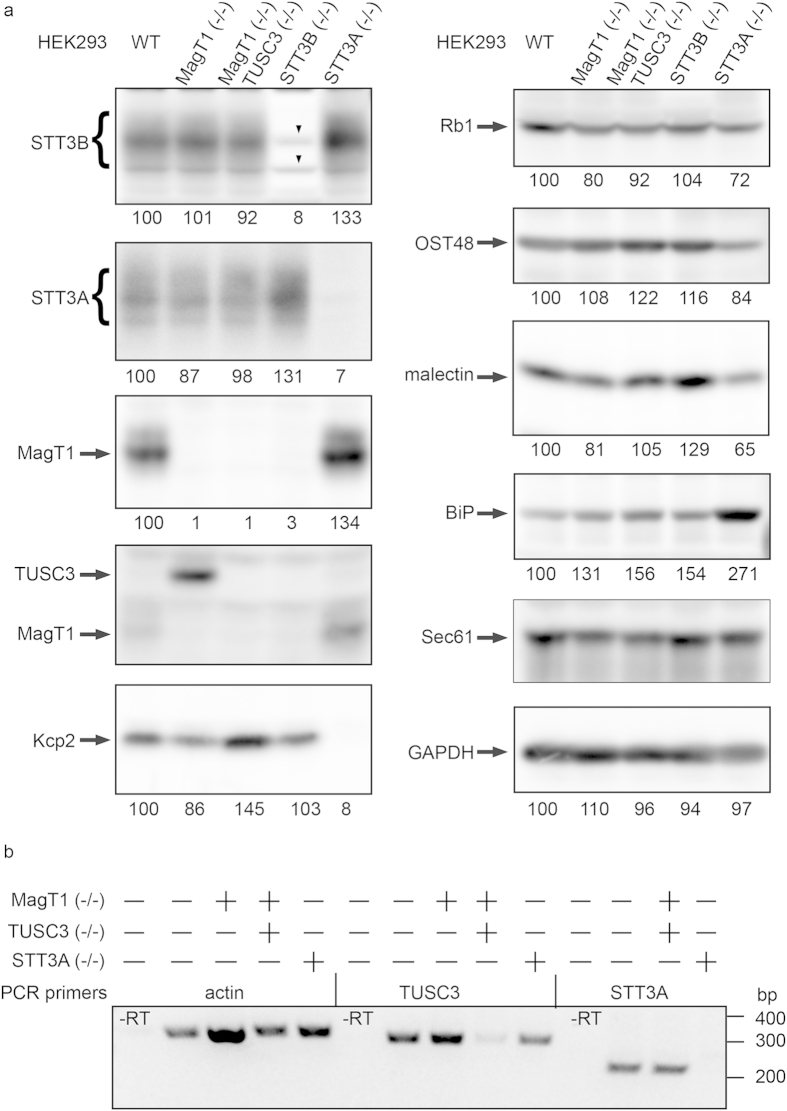
Validation of HEK293 derived cell lines. (**a**) Total protein extracts from WT or mutant HEK293 cells (50 ug) were resolved by SDS-PAGE and analyzed by protein immunoblotting using the specified sera. Downward pointing arrowheads in the STT3B blot designate non-specific background bands. Anti-TUSC3 sera cross-reacts with MagT1. Expression of the OST subunits and BiP were adjusted relative to the gel loading control (GAPDH), and were normalized to the relevant wild type control to compare the relative expression of OST subunits. (**b**) Actin, TUSC3 and STT3A mRNA expression in knockout HEK293 cells was examined by reverse transcription PCR. Amplification products were resolved on 1.5% agarose gels. Immunoblot images and agarose gel were cropped to display the region of interest. Full-length immunoblot images for panel (**a**) and the full length agarose gel image for panel (**b**) are shown in Supplemental Fig. S4.

Total cell extracts were prepared from wild type and mutant cells and resolved by SDS-PAGE for protein immunoblot analysis using antibodies raised against the OST subunits (Fig. 3a). Glyceraldehyde 3’phosphate dehydrogenase (GAPDH) was used as a protein loading control to normalize the calculated expression values for the OST subunits. Calculated expression values below 10% in the knockout cell lines represent non-specific background. Consistent with the DNA sequence analysis, the diffusely migrating STT3A and STT3B proteins are absent in the STT3A(−/−) are STT3B(−/−) cell lines, respectively. We observed a 30% increase in STT3A expression in the STT3B(−/−) cell line and a 30% increase in expression of STT3B and MagT1 in the STT3A(−/−) cells. These increases in expression level are consistent with our previous observations for siRNA treated HeLa cells^5^,^11^, and are likely explained by competition during OST assembly in wild type cells between STT3A and STT3B for a limited quantity of one or more of the shared OST subunits (Rb1, Rb2, OST48, DAD1 or OST4). The STT3A specific accessory subunit KCP2 was undetectable in extracts prepared from STT3A(−/−) cells. Likewise, the STT3B complex specific accessory subunits MagT1 and TUSC3 were not detected in STT3B(−/−) cells. We conclude that the accessory subunits (KCP2, MagT1 and TUSC3) are degraded when their cognate catalytic subunit is absent.

The complete absence of MagT1 immunoreactivity in the MagT1(−/−) cells was accompanied by increased stable expression of the TUSC3 protein (Fig. 3a) as observed for MagT1 siRNA treated HEK293 cells (Fig. 1b). MagT1 and TUSC3 compete for a shared binding site in the STT3B complex^5^, hence when MagT1 is absent TUSC3 is able to occupy the vacant binding site. It is not clear why we detect elevated amounts of KCP2 in the MagT1(−/−) TUSC3(−/−) cell line, as we do not detect incorporation of KCP2 into the STT3B complex as assayed by blue native gel electrophoresis (data not shown).

The morphology and growth rate of the knockout cell lines was indistinguishable from the parental HEK293 cells during roughly 25 passages after the clones were isolated. Hence, loss of the STT3A or STT3B complex is tolerated in cultured cells. Importantly, fibroblasts obtained from the STT3A-CDG and STT3B-CDG patients showed reductions in STT3A and STT3B protein expression respectively, and displayed glycosylation defects that were similar to siRNA treated HeLa cells^12^.

Hypoglycosylation of proteins in the ER causes an induction of the unfolded protein response (UPR) that can be detected by increased expression of lumenal chaperones including BiP^36^. BiP expression was elevated 2.7 fold in the STT3A(−/−) cell line relative to control HEK293 cells. BiP expression was elevated by 1.5 fold when the STT3B complex is either missing or lacks both MagT1 and TUSC3. The greater induction of BiP expression in STT3A(−/−) cells relative to STT3B(−/−) cells indicates that loss of the STT3A complex has a more profound impact upon glycoprotein folding in the endoplasmic reticulum likely reflecting the proportion of acceptor sites that are cotranslationally glycosylated by the STT3A complex in wild type cells.

Reverse transcription PCR was performed to evaluate mRNA levels for TUSC3 and STT3A in the knockout cell lines (Fig. 3b). RT-PCR amplification of TUSC3 mRNA in MagT1(−/−) TUSC3(−/−) cell line and STT3A mRNA in the STT3A (−/−) cell line resulted in very low yields of PCR product likely due to degradation of the mRNA transcripts by the nonsense mediated mRNA decay pathway. These results support the conclusion that STT3A(−/−) and MagT1(−/−) TUSC3(−/−) cell lines are bona-fide null cell lines. Despite the low expression of TUSC3 protein in HEK293 cells, the TUSC3 mRNA is present at comparable levels in all cell lines except the MagT1(−/−) TUSC3(−/−) cell line. Thus, the observed increase of TUSC3 protein level in the MagT1 (−/−) cell line (Fig. 3a) is not explained by enhanced transcription of the TUSC3 mRNA, but is instead explained by enhanced stability of the TUSC3 protein upon incorporation into the STT3B complex. Even though TUSC3 and MagT1 are homologues, incorporation of MagT1 into the STT3B complex is strongly favored relative to TUSC3.

### Glycosylation of proteins in knockout cell lines

**Figure 4:**
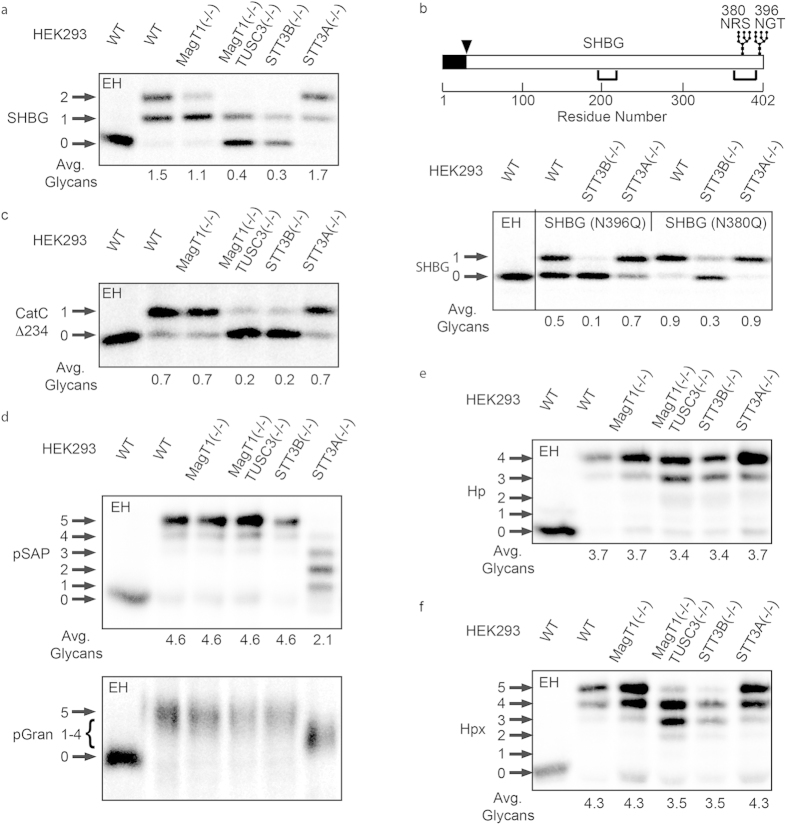
Hypoglycosylation of proteins in knockout HEK293 cell lines. Cell lines were transfected with expression vectors for the following human glycoproteins: (**a**) SHBG, (**b**) SHBG derivatives (see diagram) with single glycosylation sites (SHBG N380Q and N396Q), (**c**) a cathepsin C derivative with a single glycosylation site (CatCΔ234-HA), (**d**) prosaposin (pSAP-DDKHis), (**e**) haptoglobin (Hp-DDKHis), (**f**) hemopexin (Hpx-DDKHis). Endogenous progranulin (pGran) (**d**) was analyzed using non-transfected cells. The cells were pulse labeled for 10 min and chased for 10 min (pSAP, pGran and CatCΔ234-HA) or pulse labeled for 5 min and chased for 20 min (Hp, SHBG and Hpx). Glycoproteins precipitated with anti-DDK, anti-HA or anti-SHBG were resolved by PAGE in SDS. EH designates treatment with endoglycosidase H. Quantified values below gel lanes (**a**–**f**) are for the displayed image, which is representative of two or more experiments. (**b**) The vertical line indicates the excision of three intervening gel lanes. (**d**) Resolution of pGran glycoforms is not sufficient for quantification. Nonglycosylated forms of pSAP, Hp, and Hpx that comigrate with the EH-digested form of the substrate in HEK293 cells correspond to a non-translocated precursor and were not used to calculate the average number of glycans. (**a**–**f**) Phosphorimages were cropped to display the region of interest. Full-length phosphorimages are shown in Supplemental Fig. S5.

The knockout cell lines were transiently transfected with expression vectors for a panel of glycoproteins that contain previously characterized STT3A and STT3B dependent glycosylation sites. Pulse-chase labeling of SHBG in HEK293 cells yields a mixture of monoglycosylated SHBG and diglycosylated SHBG (Fig. 4a) as observed previously for other cell lines and in human sera^24^,^37^. The absence of both oxidoreductase subunits (MagT1(−/−) TUSC3(−/−)), or elimination of STT3B, causes a dramatic reduction in SHBG glycosylation. Single glycosylation sites mutants of SHBG were analyzed to determine which site is partially glycosylated in the STT3B(−/−) cell line (Fig. 4b). Glycosylation of the N_380_RS site (N396Q mutant) was incomplete in wild type cells and strongly inhibited in STT3B(−/−) cells. Glycosylation of the N_396_GT site (N380Qmutant) was efficient in wild type cells, but not completely blocked in STT3B(−/−) cells. Taken together with previous results^24^, we can conclude that the frequency of acceptor site skipping of SHBG sequons by the STT3A complex is influenced by sequon type (NXS sites are skipped more frequently than NXT sites). The efficiency of posttranslational glycosylation by the STT3B complex is also higher for NXT sites than NXS sites, consistent with our bioinformatic analysis of extreme C-terminal sites in the murine glycoproteome^24^. OST assays using synthetic peptide acceptors indicate that the OST has a higher affinity for NXT sequons than NXS sequons^38^.

Depletion of STT3B or MagT1 reduces glycosylation of a single acceptor site in cathepsin C^5^,^11^. The STT3B-dependent N_29_CT site in cathepsin C (pCatC) is four residues from the signal sequence cleavage site and contains a cysteine that is disulfide-bonded in the mature protein. Previously, we showed that the signal sequence proximal location of the N_29_CT site and the internal cysteine residue are both required for STT3B and MagT1 dependence in HeLa cells^5^. Glycosylation of a pCatC derivative (pCatCΔ234) that has a single acceptor site (N_29_CT) was strongly inhibited in cells lacking either STT3B or both oxidoreductase subunits (Fig. 4c).

Prosaposin and progranulin are cysteine-rich glycoproteins that are composed of four (pSAP) or seven (pGran) small repeat domains separated by spacer segments. Glycosylation of prosaposin and progranulin was greatly reduced in STT3A (−/−) cells but not reduced when cells lack the STT3B complex (Fig. 4d), consistent with previous results obtained using siRNA treated HeLa cells^11^.

Glycosylation of certain acceptor sites was not reduced in HeLa cells that were treated with STT3A or STT3B siRNA. For example, glycosylation of haptoglobin (Hp) is insensitive to knockdown of STT3A^25^. A single acceptor site in haptoglobin (N_241_YS) was reduced by treatment with the STT3B siRNA^25^. The insensitivity of the haptoglobin sites to siRNA mediated depletion of STT3A or STT3B might indicate that the Hp sequons can be efficiently glycosylated by either STT3A or STT3B, or it might be explained by the incomplete depletion of STT3A and STT3B that is achieved by siRNA treatment. Pulse labeling of haptoglobin in the STT3B(−/−) and MagT1(−/−) TUSC3(−/−) cell lines caused less than a 10% reduction in glycan occupancy (Fig. 4e).

Two of the five glycosylation sites in hemopexin (Hpx) show intermediate STT3B-dependence due to the presence of an internal cysteine residue (N_187_CS) or a C-terminal location (N_453_VT)^25^. Glycosylation of Hpx was not reduced in the STT3A(−/−) cell line, but was reduced by 20% in the STT3B(−/−) and MagT1(−/−)TUSC3(−/−) cell lines (Fig. 4f). The accumulation of glycoforms that lack one or two glycans is consistent with the presence of two acceptor sites that show partial STT3B dependence. The other three sites in hemopexin can be glycosylated by either STT3A or STT3B.

The results obtained by pulse-labeling of the knockout cell lines (Fig. 4) were compared to results obtained with siRNA treated HEK293 cells (Fig. 1) or siRNA treated HeLa cells^5^,^11^,^24^,^25^ to evaluate the utility of the knockout cell lines (Table 1). Several conclusions can be drawn from the data in Table 1. Glycosylation of a protein with previously identified STT3A dependent sites (pSAP) was more severely reduced in the STT3A(−/−) cell line than in siRNA treated HEK293 or HeLa cells. On average, the STT3B complex can modify two of the five sites in pSAP when the STT3A complex is absent. The heterogeneity of pSAP glycoforms in STT3A(−/−) cells suggests that STT3B complex does not have a strong preference for a subset of the five acceptor sequons. Glycosylation of the previously identified STT3B dependent sites in SHBG and pCatCΔ234 was more severely reduced in STT3B(−/−) cells than in siRNA treated HeLa or HEK293 cells. Based upon the residual glycosylation of these three sites in STT3B(−/−) cells, acceptor site skipping by STT3A can approach 100%. Analysis of haptoglobin and hemopexin glycosylation in STT3B(−/−) or STT3A(−/−) cells yielded results that were quite similar to what we obtained with siRNA treated HeLa cells. Analysis of Hp and Hpx glycosylation provides strong evidence for a class of glycosylation sites that can be glycosylated by either OST complex with high efficiency. A more global analysis of acceptor site occupancy in STT3A (−/−) and STT3B(−/−) cells will be required to identify additional features that mandate glycosylation by one of the two OST complexes.

Consistent with our previous analysis^5^, STT3B complexes that lack an oxidoreductase subunit have little or no residual glycosylation activity, at least with the substrates we have analyzed to date. The oxidoreductase subunit in the STT3B complex is thought to form mixed disulfides with glycoprotein substrates explaining why MagT1 is necessary for glycosylation of sequons with an internal cysteine residue (e.g., N_29_CT sequon in pCatC). However, the oxidoreductase subunits are also required for glycosylation of the extreme C-terminal sequons in a SHBG derivative that lacks the C-terminal disulfide^5^, suggesting that MagT1 or TUSC3 are needed for substrate recognition. The MagT1(−/−) cells had a less severe defect in glycosylation of STT3B dependent substrates than MagT1 siRNA treated HeLa cells due to enhanced stable expression of TUSC3 in the MagT1 deficient HEK293 cells. In contrast, the MagT1(−/−)TUSC3(−/−) HEK293 cells had a more severe glycosylation defect that MagT1 siRNA treated HeLa or HEK293 cells. We have not identified a substrate that has a specific requirement for one of the two oxidoreductases, suggesting that these gene products are functionally redundant. An overlapping function of MagT1 and TUSC3 would help explain why TUSC3^21^,^22^,^23^ or MagT1^19^ deficient human patients have less severe symptoms than the STT3B-CDG patient^12^.

### Restoration of glycosylation in knockout cell lines

**Figure 5:**
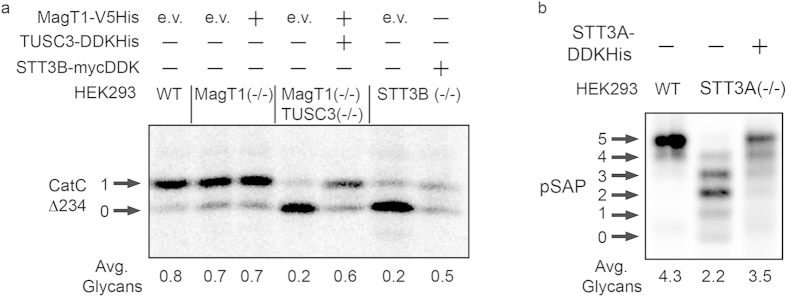
Restoration of N-glycosylation of proteins in knockout cell lines. (**a**) Wild type or mutant HEK293 cells were cotransfected with an OST subunit expression vector (empty vector (e.v.), MagT1-V5 His, STT3B-myc DDK, or MagT1-V5 His plus TUSC3-DDKHis) and an expression vector for CatCΔ234-HA. CatCΔ234-HA was immunoprecipitated with anti-HA sera from total cell extracts after a 10 min pulse, 10 min chase labeling period. (**b**) Wild type and mutant HEK293 cells were transfected with an OST expression vector (empty vector or STT3A-DDKHis). After 48 h of culture the cells were cotransfected with the OST expression plasmid and a pSAP-DDKHis expression vector. Glycosylation of pSAP-DDKHis was evaluated by a 10 min pulse, 10 min chase labeling procedure 24 h after the second transfection. pSAP-DDKHis was immunoprecipitated with anti-DDK sera. Phosphorimages were cropped to display the region of interest. Full-length phosphorimages are shown in in Fig. S6.

The HEK293 derived knockout cell lines were cotransfected with a previously characterized expression plasmid for an OST subunit^5^,^27^ and a glycoprotein reporter to determine whether normal glycosylation could be restored by transient expression of the missing OST subunit (Fig. 5). As expected, expression of MagT1-V5His had no impact on glycosylation of pCatCΔ234 in the MagT1(−/−) cells, since the enhanced stable expression of TUSC3 mitigates any glycosylation defect caused by loss of MagT1 (Fig. 5a). More importantly, coexpression of MagT1-V5His and TUSC3-DDKHis in the (MagT1(−/−)TUSC3(−/−) mutant allows near-normal glycosylation of pCatCΔ234 (Fig. 5a). Expression of STT3B-mycDDK in the STT3B(−/−) cell line was somewhat less effective in restoring normal glycosylation of pCatCΔ234 (Fig. 5a). Although we observed lower incorporation of radiolabel into both pCatCΔ234 glycoforms in the cells expressing STT3B-mycDDK in this experiment, it is the ratio of the two glycoforms rather than total pCatCΔ234 synthesis that is used to evaluate the efficiency of glycosylation. Expression of STT3A-DDKHis in the STT3A(−/−) cell line improved, but did not fully restore glycosylation of prosaposin (Fig. 5b). Restoration of glycosylation by expression of STT3A or STT3B in the null cell lines is a more complicated process, because it entails de novo assembly of a seven to eight subunit integral membrane protein. Moreover, the exogenously expressed catalytic subunit (e.g. STT3B-mycDDK) will compete with newly synthesized endogenous STT3A for assembly with the shared subunits (Rb-1, Rb-2, OST48, DAD1 and OST4) of the STT3A and STT3B complexes.

The HEK293 derived null cell lines described here will be an important tool for further analysis of OST complex function in mammalian cells. While the results obtained with the knockout cell lines validates our previous conclusions about STT3A and STT3B dependent glycosylation sites, the interpretation of results was greatly simplified in the null cell lines relative to the siRNA treated cells. The null cell lines will be useful for experimental approaches where transient siRNA mediated knockdowns are not optimal including generation of cells with deficiencies in two or more proteins. These HEK293 derived null cell lines should also prove useful for mass spectrometric analysis of sequon occupancy of glycoproteins with multiple glycosylation sites like prosaposin or progranulin. The null cell lines are good models for complementation experiments where wild type and mutant alleles of an OST subunit will be tested for the ability to restore glycosylation to the mutant cells. The latter experiments are more challenging in siRNA treated cells due to the incomplete depletion of the genome-encoded wild type subunit and the need to generate an expression construct that is insensitive to the siRNA.

## Materials and Methods

### Cell culture, siRNA transfection and protein expression

HEK293 cells (ATCC CRL-1573) were cultured in 10 cm^2^ dishes at 37°C in DMEM (GIBCO), 10% fetal bovine serum (GIBCO, 10437-028) with penicillin (100 units/ml) and streptomycin (100 μg/ml). Cells were seeded at 30% confluency for siRNA transfection or 80% confluency for plasmid transfection in 60 mm dishes and grown for 24 h prior to transfection with siRNA (NC-60 nM; STT3A-50 nM; MagT1-50 nM; TUSC3-50 nM, STT3B-60 nM) or plasmid (8 μg) and Lipofectamine 2000 in Opti-MEM (GIBCO) using a protocol from the manufacturer (Invitrogen). Plasmid transfection was performed after 48 h of siRNA transfection and cells were assayed 24 h later. The siRNAs designed to knockdown STT3A, STT3B, MagT1 and TUSC3 were described previously^5^,^11^. The siRNA duplexes were purchased from Sigma Aldrich.

### Generation of HEK293 derived cell lines

The CRISPR/Cas9 system was used to generate HEK293 derived cell lines using the protocols described by the Zhang laboratory^39^. Design of the guide RNAs was carried out using the CRISPR design tool (http://crispr.mit.edu)^40^. Guide oligonucleotide primers were cloned into the vector pSpCas9(BB)-2A-GFP (Addgene plasmid ID: PX458). HEK293 cells were cultured in 60 mm dishes and transfected with 5 μg of the CRISPR plasmids. Cells were collected by trypsinization 24 h after transfection and subjected to FACS using a BD FACSVantage DV1 Cell Sorter to obtain GFP positive cells that were plated as single cells into 96 well plates. After two weeks, surviving colonies were isolated and expanded. Total cell extracts were prepared from colonies for protein immunoblot analysis using antibodies specific for the targeted protein (STT3A, STT3B, MagT1 or TUSC3). Anti-MagT1 sera was used as a secondary screen for potential STT3B-null clones, as MagT1 levels drop in cells treated with STT3B siRNA^5^. Anti-TUSC3 sera was used as a secondary screen for MagT1-null clones.

The genomic DNA was harvested with the Quick Extract Solution (EpiCentre). Primers for PCR amplification of genomic DNA flanking the CRISPR target sites were designed using PRIMER BLAST (NCBI). Genomic fragments were amplified with Phusion or Q5 High Fidelity polymerases (New England Biolabs). 400 ng of PCR products were re-hybridyzed and incubated with T7E1 endonuclease (New England Biolabs) for 20 min at 37 °C. DNA fragments were separated on a 2% agarose gel and quantified using Image Quant TL software (GE Scientific). Amplicons were cloned into pJet1.2 vector (Thermo Scientific) for DNA sequencing. Sequences were aligned to the wild type DNA to observe insertions or deletions. A bioinformatic tool at http://crispr.mit.edu was used to search for potential off-target sites for each of the sgRNAs (Supplementary Table S2). DNA flanking potential off target sites was amplified by PCR and evaluated using T7E1 endonuclease digestions (Supplementary Fig. S1).

### Reverse transcription PCR

Total RNA was purified from HEK293 cells using TRIzol reagent (Invitrogen) according to the manufacturer’s instructions. Total RNA (1 μg) was used for a reverse transcription reaction with a Superscript IV kit (Invitrogen) and an oligo dT primer. The RT products (0.25–2.5 μl) were amplified by PCR.

### Expression vectors

The STT3B-myc-DDK expression vector was purchased from OriGene. Plasmids encoding MagT1-V5His and TUSC3-DDKHis^5^, SHBG^37^, pCatCΔ234-HA^11^, haptoglobin (Hp-DDKHis), hemopexin (Hpx-DDKHis), SHBG N380Q and N396Q^24^, pSAP-DDKHis^27^ and STT3A-DDKHis^12^ have been described previously.

### Antibodies and protein immunoblotting

Rabbit polyclonal antibodies specific for OST subunits (anti-STT3B^11^, anti-STT3A^4^, ribophorin I (RIC6^41^) and OST48^42^) and the C-terminus of Sec61^43^ were characterized in previous publications. Antibodies to MagT1 (17430), TUSC3 (16039), GAPDH (60004) were obtained from Proteintech Group, Inc. Rabbit polyclonal anti-KCP2 (LS-C187087/55105) was from LifeSpan BioSciences. Mouse monoclonal anti-BiP (610978) was from BD Biosciences. Goat polyclonal antisera specific for cathepsin C (AF1071), progranulin (AF2420) and SHBG (VFJ01) were purchased from R&D Systems. Anti-malectin (HPA007538) and rabbit IgG reagent (I-5006) were obtained from Sigma-Aldrich. The following antibodies against epitope tags were used: anti-HA (11867423001, Roche), anti-DDK (F3165 anti-FLAG M2, Sigma).

Expression of STT3B, MagT1, KCP2, malectin, ribophorin I, Sec61, GAPDH and BiP in cells was analyzed by protein immunoblotting as described previously^5^. The band intensities were calculated using Image Quant TL software (GE scientific) and normalized to the wild type level.

### Pulse-chase radiolabeling and immunoprecipitation

Glycoprotein substrates in HEK293 cells were pulse-chase labeled with Tran^35^S label (Perkin Elmer) as described previously^5^. As indicated, immunoprecipitated proteins were digested with endoglycosidase H (New England Biolabs). Dry gels were exposed to a phosphor screen (Fujifilm), scanned in Typhoon FLA 9000 and quantified using Image Quant TL software (GE scientific).

## Additional Information

**How to cite this article**: Cherepanova, N. A. and Gilmore, R. Mammalian cells lacking either the cotranslational or posttranslocational oligosaccharyltransferase complex display substrate-dependent defects in asparagine linked glycosylation. *Sci. Rep.* **6**, 20946; doi: 10.1038/srep20946 (2016).

## Supplementary Material

Supplementary Information

## Figures and Tables

**Table 1:** Glycosylation of substrates in siRNA-treated or knockout cells.

Substrates (sequons)	Control			MagT1			MagT1, TUSC3	STT3A			STT3B		
	NC siRNA		WT	siRNA		KO	KO	siRNA		KO	siRNA		KO
	HeLa	HEK	HEK	HeLa	HEK	HEK	HEK	HeLa	HEK	HEK	HeLa	HEK	HEK
pSAP (5)	4.7	4.5	4.6	4.7	4.2	4.6	4.6	3.4	3.6	2.1	4.7	4.2	4.6
Hp (4)	3.5	nd	3.7	nd	nd	3.7	3.4	3.5	nd	3.7	3.4	nd	3.4
CatC-Δ234 (1)	0.9	nd	0.7	0.6	nd	0.7	0.2	0.9	nd	0.7	0.6	nd	0.2
Hpx (5)	4.5–4.7	nd	4.3	4.0	nd	4.3	3.5	4.7	nd	4.3	3.8	nd	3.5
SHBG (2)	1.4	1.6	1.5	1.0	1.5	1.1	0.4	1.5	1.5	1.7	1.0	1.3	0.3
SHBG N396Q (1)	0.7	nd	0.5	nd	nd	nd	nd	0.7	nd	0.7	0.3	nd	0.1
SHBG N380Q (1)	1.0	nd	0.9	nd	nd	nd	nd	0.9	nd	0.9	0.8	nd	0.3

Average glycans/protein for STT3A- and STT3B-dependent substrates determined in wild type (WT) and knockout (KO) HEK293 cell lines or in wild type HeLa or HEK293 cells treated with negative control (NC) siRNA or siRNAs specific for MagT1, STT3A or STT3B. HEK293 is abbreviated as HEK. Values shown are the average of two or more determinations. The abbreviation nd indicates not determined.
