# Supplementary material for: Mammalian cells lacking either the cotranslational or posttranslocational oligosaccharyltransferase complex display substrate-dependent defects in asparagine linked glycosylation
Source: Sci Rep. 2016 Feb 11;6:20946. doi: 10.1038/srep20946 (PMC4750078; doi:10.1038/srep20946)
Supplement: Supplementary Information [file srep20946-s1.pdf]

Mammalian cells lacking either the cotranslational or posttranslocational oligosaccharyltransferase complex display substrate-dependent defects in asparagine linked glycosylation

Natalia A. Cherepanova and Reid Gilmore

Supplemental Materials

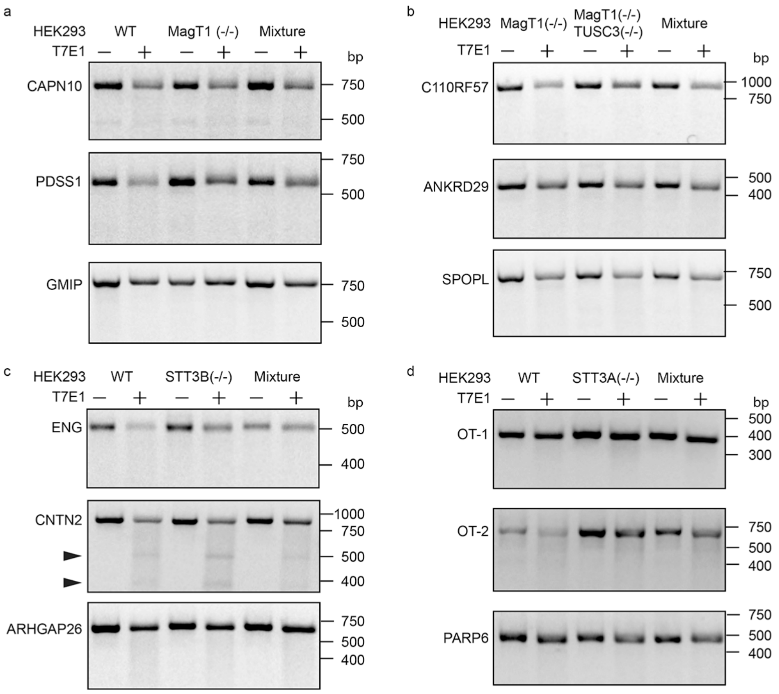

Figure S1. T7E1 analysis of the most probable off-target sites in the following cell lines: (**a**) the MagT1(-/-) HEK293 cells; (**b**) MagT1(-/-) TUSC3(-/-) HEK293 cells, (**c**) STT3B(-/-) HEK293 cellsor (**d**) STT3A(-/-) HEK293 cells. Potential off target sites are listed in Supplementary Table S2. PCR products from wild type and mutant cell lines were amplified, rehybridized (± WT PCR products) and subjected to cleavage with T7E1 endonuclease. Cleavage fragments were analysed on 2.0% agarose gel. Labeled arrows in panel **c** designate T7E1 cleavage products observed in the CNTN2 gene target in both wild type and mutant cell lines indicative of heterozygosity at this locus.

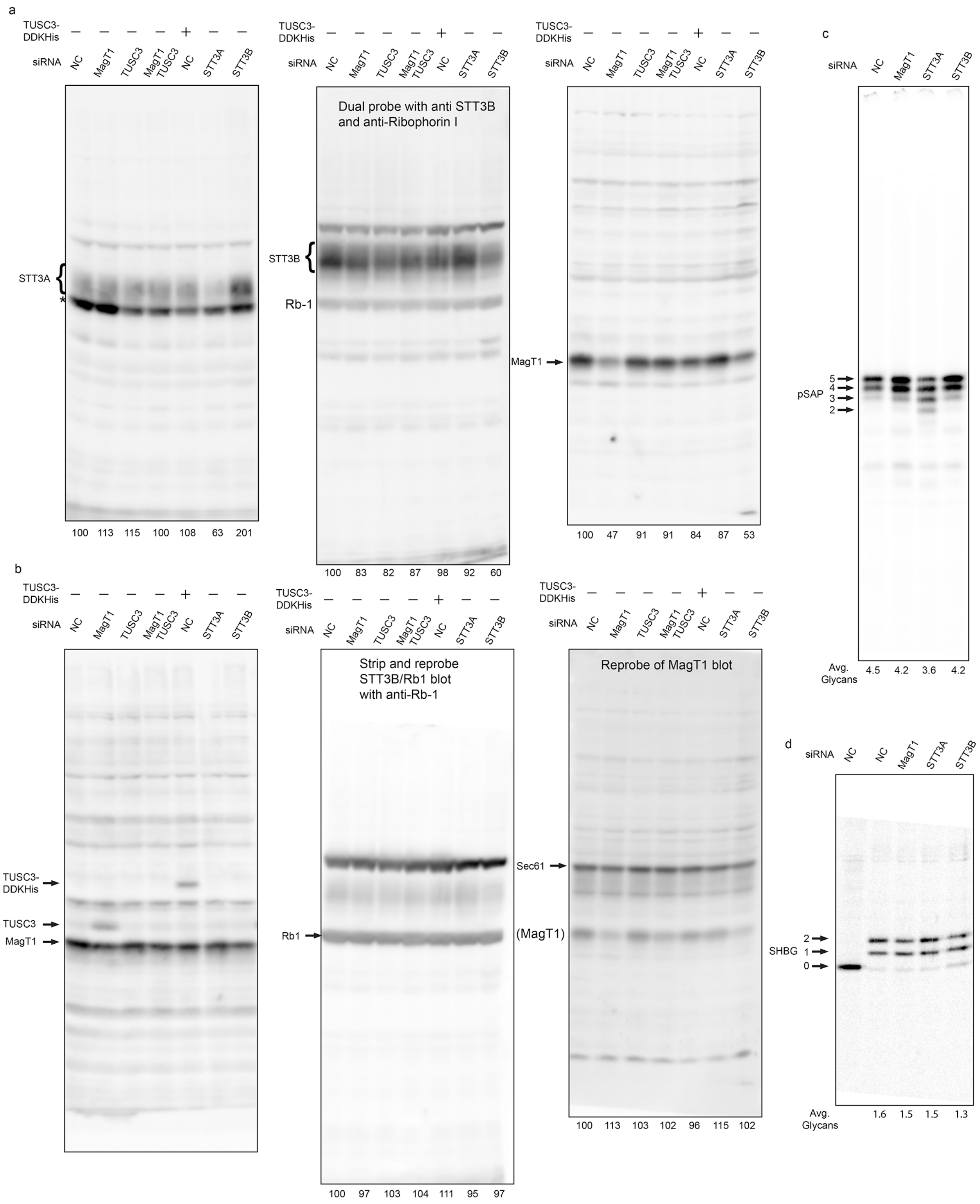

Figure S2. (**a**,**b**) Full-length blot images for Fig. 1**a** and Fig 1**b**. As indicated blots were simultaneously probed with two antisera, or reprobed with a second antibody. (**c**,**d**) Full-length phosphorimages of the gels for Fig. 1**c** and Fig. 1**d**.

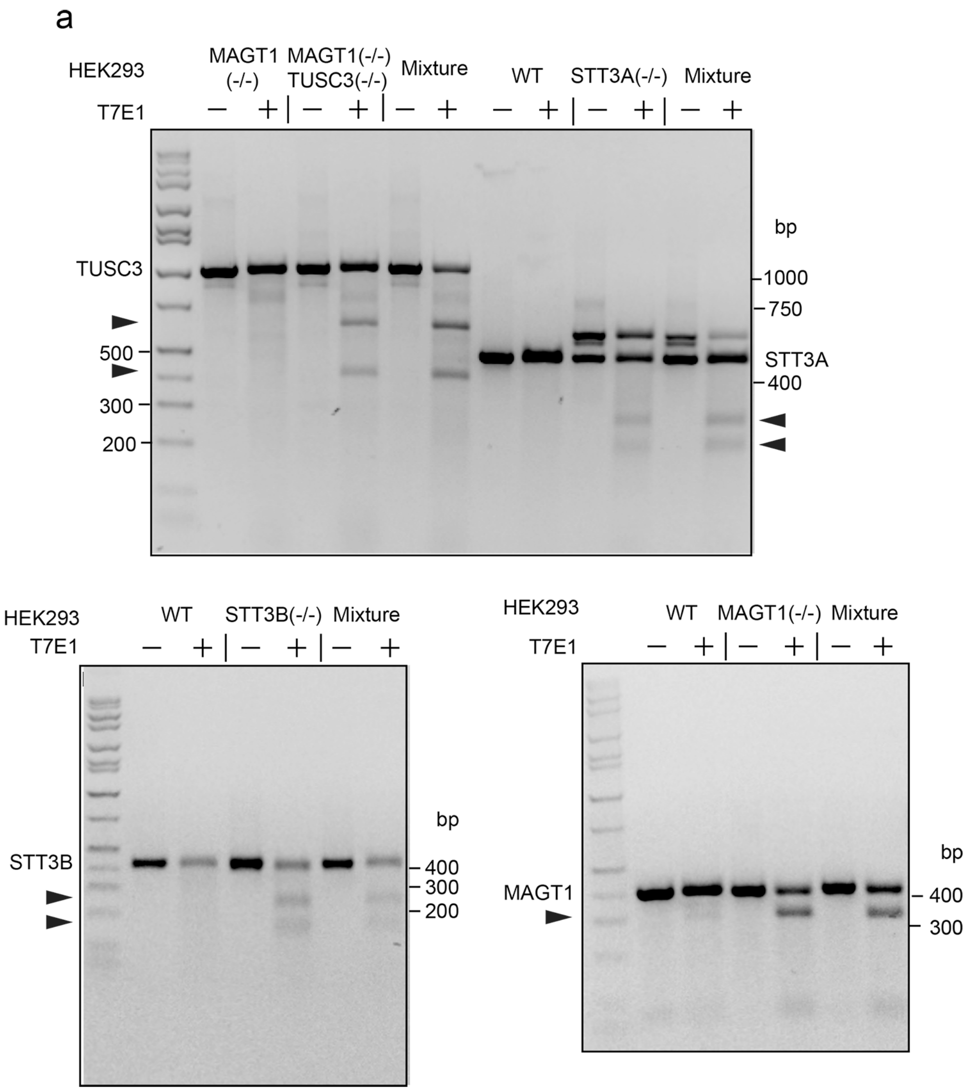

Figure S3. Full-length gel images for the T7 endonuclease assays shown in Figure 2**a**. Molecular weight markers were electrophoresed in the left hand lane.

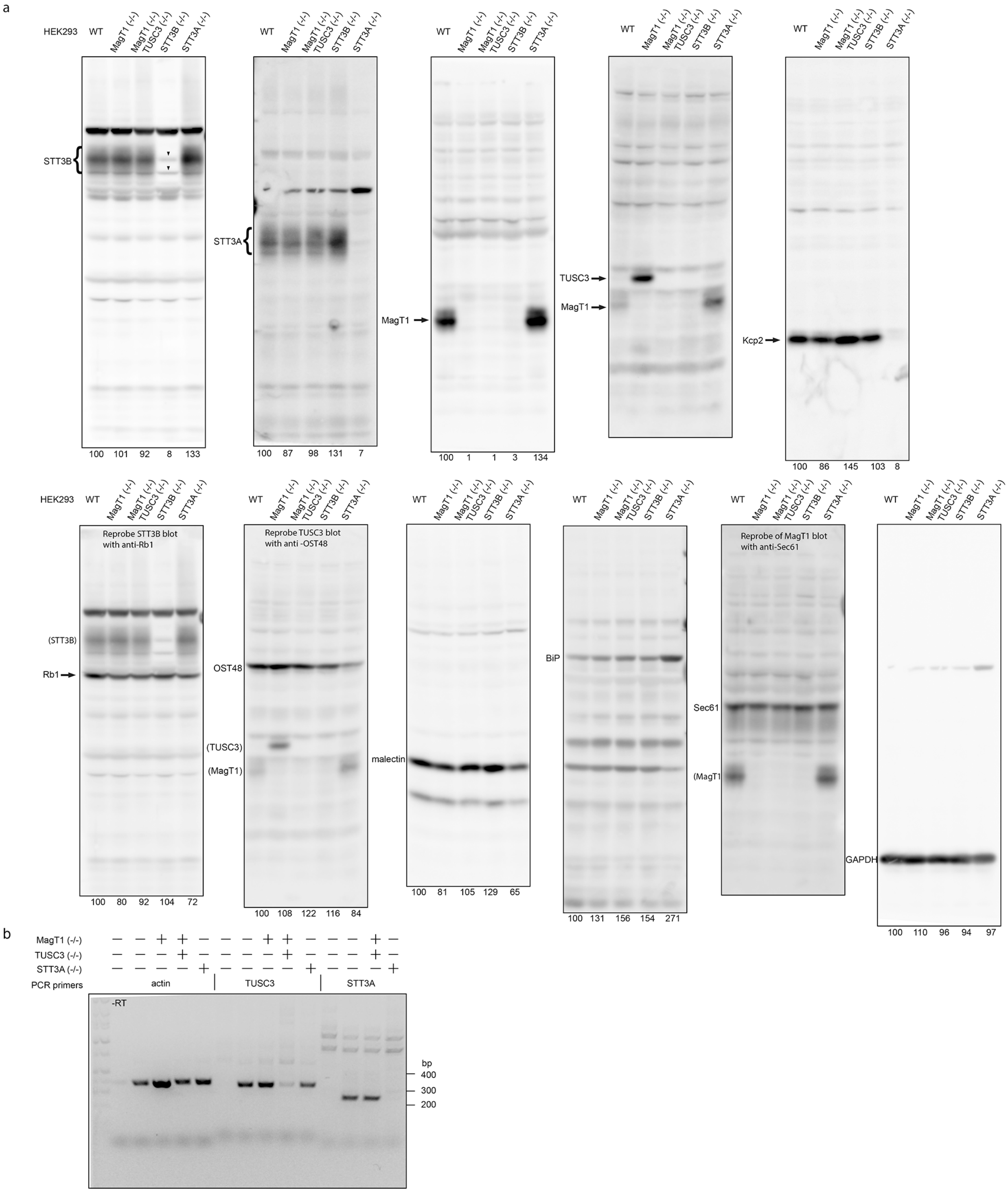

Fig. S4 (**a**) Full-length immunoblot images for Fig. 3**a**. As indicated, several immunoblots were reprobed with a second antibody. (**b**) Full-length agarose gel image for the experiments shown in Fig. 3**b**.

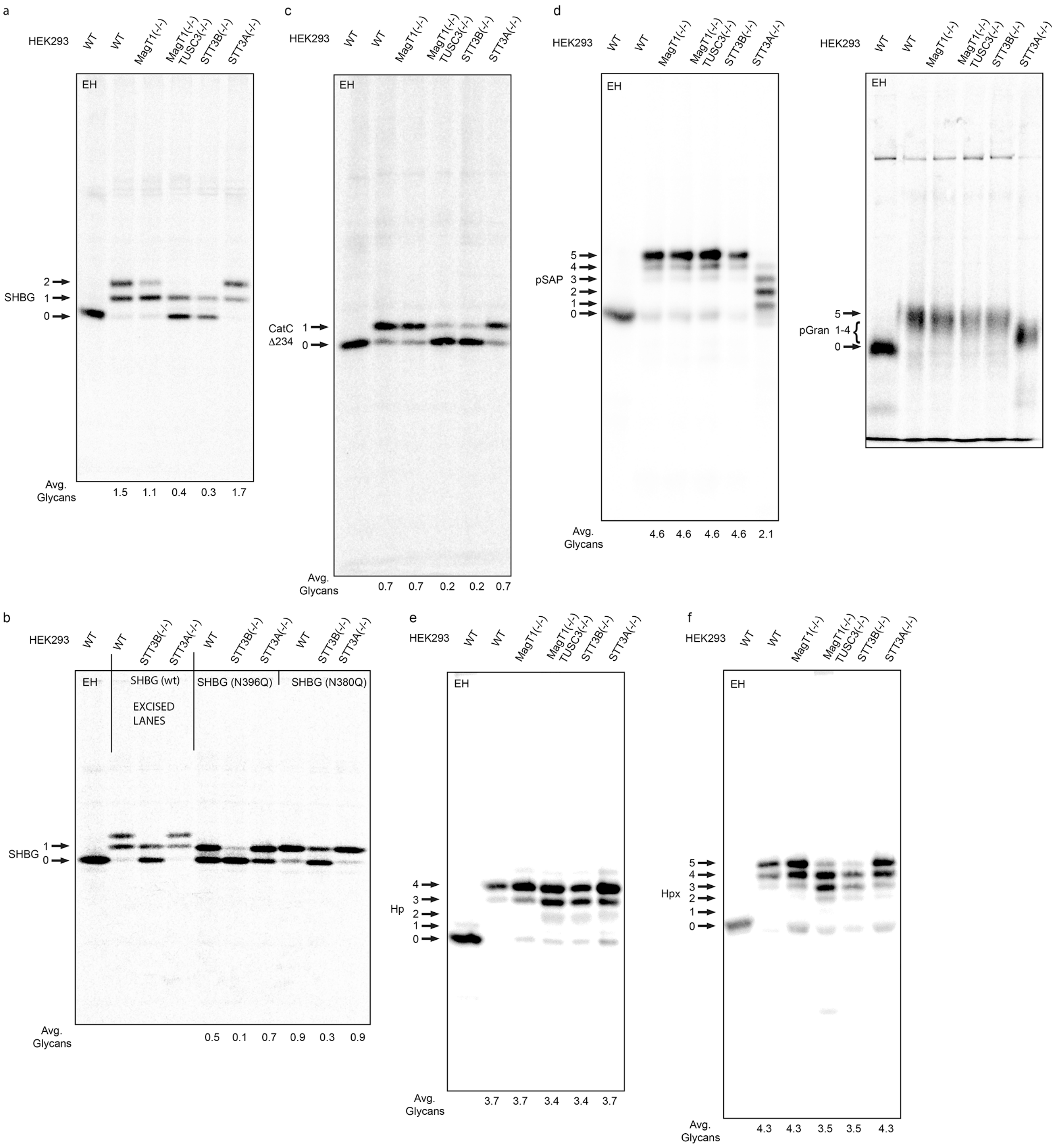

Fig. S5. Full-length phosphorimages for the gels shown in Fig. 4. (**b**) The three lanes excised from the gel image used for Fig. 4**b** are replicates of lanes shown in Fig. 4a.

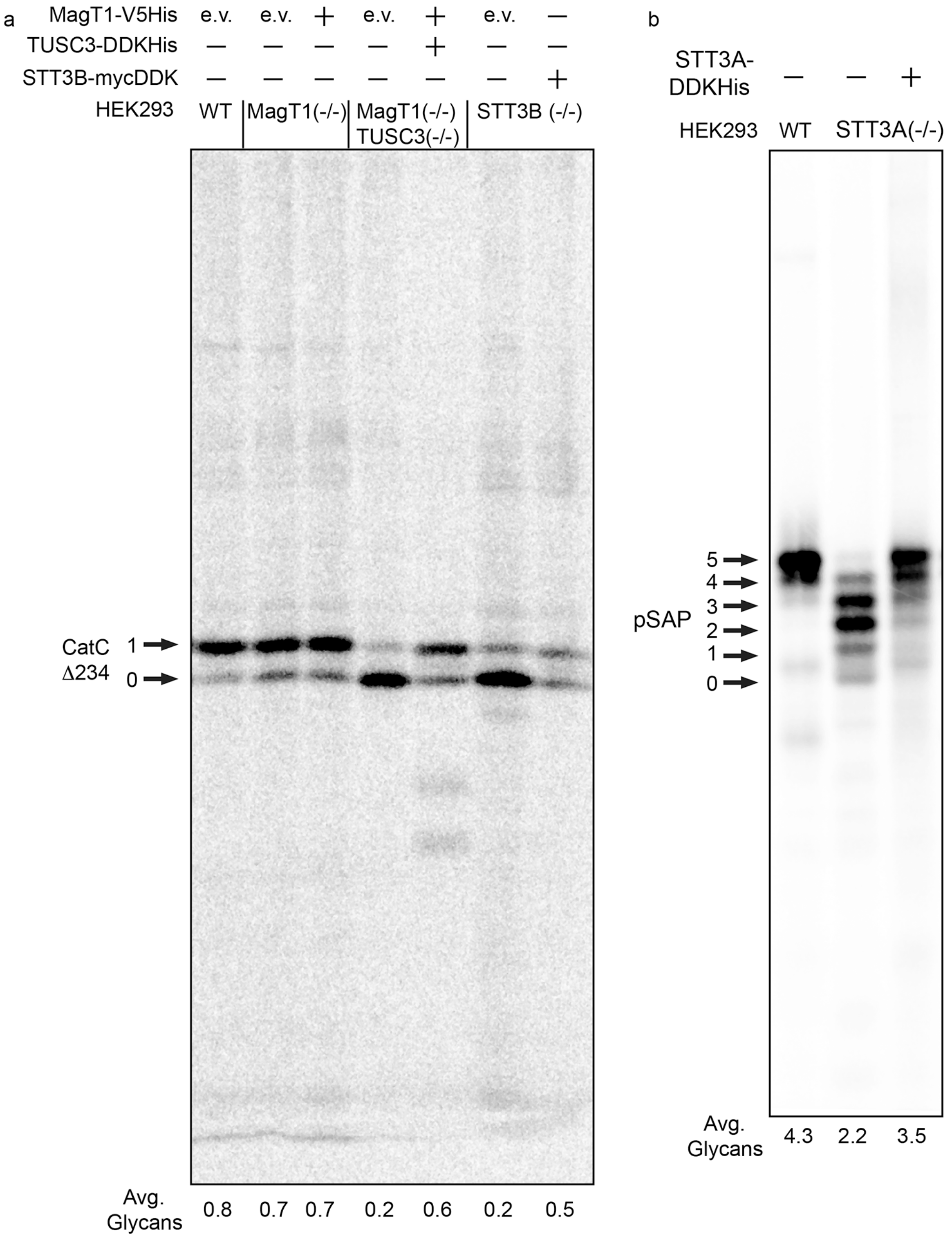

Fig. S6. Full-length phosphorimages for the gels shown in Fig. 5.

Table S1. Selected target sequences

	sequence	score	orientation	exon
MagT1 target 1*	CGAACATGGCAGCGCGTTGG**CGG**	95	sense	1
MagT1 target 2	TGGCAGCGCGTTGGCGGTTT**TGG**	94	sense	1
MagT1 target 3	AGTTCTGTCGGCGATCCACC**GGG**	94	antisense	4
STT3B target 1	CGCCGCCTTGTGCGCGCACT**GGG**	95	antisense	1
STT3B target 2*	CTGAGCATCAACCTACGACT**TGG**	96	sense	9
STT3B target 3	CACTCCACGGGGACGAGTTG**AGG**	93	antisense	1
TUSC3 target 1*	CATTCGGAAGATTGAGCGTC**TGG**	94	antisense	1
TUSC3 target 2	GCCCTGTTAGTGTCGCTTGT**TGG**	92	sense	4
STT3A target 1	TACCCTTTGGGACGAATCAT**TGG**	92	sense	3
STT3A target 2	GGTAAGGTGGTACGTGACGA**TGG**	95	antisense	4
STT3A target 3*	ATACCCATATTTCTCGAGTA**GGG**	89	sense	13

*Represents the target sequence cell lines selected for further analysis. PAM sequence is shown in green

Table S2. Potential off-target sequences for sgRNAs

Target	Off-target	Mismatches	Score	Ref	Location	Gene ID
MagT1	CGAGAATGGCCGAGCGTTGGTGG	4MMs [4:5:11:13]	0.3	NM_016573	chr19:-19745655	GMIP<br>GEM interacting protein<br>Gene ID: 51291
	CAAACATGGGAGGGCGCTGGCAG	4MMs [2:10:13:17]	0.2	NM_023083	chr2:-241536058	CAPN10<br>calpain 10<br>Gene ID: 11132
	CGACCATGGCCTCGCGCTGGTGG	4MMs [4:11:12:17]	0.2	NM_014317	chr10:+26986634	PDSS1<br>prenyl (decaprenyl) diphosphate synthase, subunit 1<br>Gene ID: 23590
STT3B	CAGAGCATCCTCCTCCGACTGGG	4MMs [2:10:11:15]	0.2	NM_001114753	chr9:-130588789	ENG endoglin<br>Gene ID: 2022
	CTGAGCTTCATCTTACTACTAAG	4MMs [7:11:13:17]	0.1	NM_001135608	chr5:-142604024	ARHGAP26<br>Rho GTPase activating protein 26<br>Gene ID: 23092
	GTGAGCACCAACCTGGGACTTGG	4MMs [1:8:15:16]	0.1	NM_005076	chr1:+205039665	CNTN2<br>contactin 2 (axonal)<br>Gene ID: 6900
TUSC3	CCATCGTAAGATTGAGCCTCAAG	4MMs [2:3:7:18]	0.2	NM_001001664	chr2:+139328622	SPOPL<br>speckle-type POZ protein-like<br>Gene ID: 339745
	CATTTGAAAGATTGATCCTCCAG	4MMs [5:7:16:18]	0.0	NM_173505	chr18:+21229266	ANKRD29<br>ankyrin repeat domain 29<br>Gene ID: 147463
	CCATCGTAAGATTGAGCCTCAAG	4MMs [2:3:7:18]	0.0	NM_001001664	chr2:+139328622	C11orf57<br>chromosome 11 open reading frame 57<br>Gene ID: 55216
STT3A	GTACACAAATTTCTCGGGTATAG	4MMs [1:5:8:17]	0.6	-	chr5:-108087574	OFF1
	ATACGCATTTTTCTTGAGTATAG	3MMs [5:9:15]	0.5	-	chr4:+189441414	OFF2
	AAACGCATAGTTCACGAGTACAG	4MMs [2:5:10:14]	0.2	NM_020214	chr15:+72549749	PARP6<br>poly (ADP-ribose) polymerase family, member 6<br>Gene ID: 56965
